# Treatment with a new benzimidazole derivative bearing a pyrrolidine side chain overcomes sorafenib resistance in hepatocellular carcinoma

**DOI:** 10.1038/s41598-019-53863-2

**Published:** 2019-11-21

**Authors:** Fat-Moon Suk, Chao-Lien Liu, Ming-Hua Hsu, Yu-Ting Chuang, Jack P. Wang, Yi-Jen Liao

**Affiliations:** 1 Division of Gastroenterology, Department of Internal Medicine, Wan Fang Hospital, Taipei Medical University, Taipei, Taiwan; 2 Department of Internal Medicine, School of Medicine, College of Medicine, Taipei Medical University, Taipei, Taiwan; 3 School of Medical Laboratory Science and Biotechnology, College of Medical Science and Technology, Taipei Medical University, Taipei, Taiwan; 4 Department of Chemistry, National Changhua University of Education, Changhua, Taiwan; 5 Department of International Medicine, Taipei City Hospital Ranai Branch, Taipei, Taiwan

**Keywords:** Cancer therapeutic resistance, Hepatocellular carcinoma

## Abstract

Hepatocellular carcinoma (HCC) is a major cause of cancer-related death worldwide. Currently, sorafenib is the standard first-line drug for patients with advanced HCC. However, long-term exposure to sorafenib often results in reduced sensitivity of tumour cells to the drug, leading to acquired resistance. Therefore, developing new compounds to treat sorafenib resistance is urgently needed. Although benzimidazole and its derivatives have been reported to exert antimicrobial and antitumour effects, the anti-drug resistance potential of these molecules is still unknown. In this study, we established sorafenib-resistant (SR) cell lines and an acquired sorafenib resistance xenograft model. We showed that treatment with a benzimidazole derivative bearing a pyrrolidine side chain (compound **9a**) inhibited the proliferation of SR cells by blocking the phosphorylation of AKT, p70S6 and the downstream molecule RPS6. In addition, caspase 3/PARP-dependent apoptotic signals were induced in **9a**-treated cells. Regarding epithelial-mesenchymal transition (EMT) activities, **9a** treatment significantly suppressed the migration of SR cells. In particular, the levels of EMT-related transcription factors (snail, slug and twist) and mesenchymal markers (vimentin and N-cadherin) were downregulated. In the acquired sorafenib resistance xenograft model, compound **9a** administration decreased the growth of tumours with acquired sorafenib resistance and the expression of the HCC markers α-fetoprotein, glypican 3 and survivin. In conclusion, treatment with this compound may be a novel therapeutic strategy for patients with sorafenib resistance.

## Introduction

Liver cancer is the seventh leading cause of cancer-related death worldwide^1^. Hepatocellular carcinoma (HCC) is the most common type of liver cancer and comprises approximately 75–85% of cases. Traditional treatments such as surgical resection and liver transplantation may be useful in the early stages of disease^2^. However, for late-stage disease, sorafenib is the only first-line drug approved by the Food and Drug Administration (FDA) in the United States for systemic therapy^3,4^. The molecular pathogenesis of liver cancer, which involves numerous molecular aberrations and signalling activation, is mediated through multiple factors^5,6^. Sorafenib is an orally administered multikinase inhibitor that blocks tumour cell proliferation and angiogenesis by inhibiting serine/threonine kinases and tyrosine kinase receptors^7^. Sorafenib displays strong inhibition of the RAF/MEK/ERK signalling pathway and suppresses other tyrosine kinase receptors, such as vascular endothelial growth factor receptor, FMS-like tyrosine kinase 3 and platelet-derived growth factor receptor. Two large-scale epidemiological studies demonstrated that sorafenib significantly extended the survival of HCC patients compared with placebo-treated patients, increasing survival by nearly 3 months^8,9^. However, most patients who continuously receive sorafenib acquire resistance to this drug^10^. In addition, the relatively high cost, the lack of efficacy against metastasis, and severe drug-related adverse events limit the clinical application of sorafenib^8,9,11^. Therefore, developing new compounds for the treatment of sorafenib-resistant HCC is urgently warranted.

Benzimidazole contains a bicyclic aromatic complex comprising a fusion of imidazole and a benzene ring and is a part of vitamin B12^12,13^. Since benzimidazole is structurally analogous to purine, it can interact with other biopolymers. Therefore, this compound contains a critical pharmacophore and has medicinal chemistry drug discovery potential^14^. Benzimidazole and its derivatives have promising prospects for curing different diseases, and the relevant properties include antibacterial^15,16^, anti-tubercular^17^, anti-fungal^18^, anti-protozoal^19,20^, anti-HIV^21^, and anti-hepatitis virus activities^22,23^. Regarding anticancer activity, the heterocyclic imidazole ring structure of benzimidazole-derived scaffolds is widely used in the treatment of different cancers^24^. Benzimidazole derivatives have shown relatively strong cytotoxicity against liver, breast and cervical cancer cells^25–27^. However, the pharmacological activity of benzimidazole derivatives in drug-resistant HCC is still unknown. In this study, we established sorafenib-resistant (SR) liver cancer cell lines and an acquired sorafenib resistance xenograft model. Then, we evaluated the ability of a novel benzimidazole derivative bearing a pyrrolidine side chain (named compound **9a**) to overcome sorafenib resistance both *in vitro* and *in vivo*. We showed that compound **9a** treatment not only inhibited SR cell proliferation and migration but also induced caspase-dependent apoptotic signalling. Moreover, compound **9a** administration retarded the progression of liver cancer with acquired sorafenib resistance in the xenograft model.

## Results

### Establishment of SR liver cancer cells

**Figure 1:**
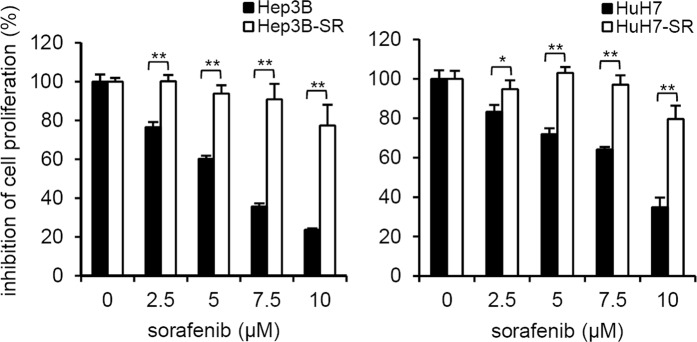
Establish sorafenib resistant liver cancer cells. Parental and sorafenib resistant (SR) HuH7 and Hep3B cells were exposed sorafenib at the indicated doses for 48 hrs and the inhibition of cell proliferation was analyzed using a MTT assay kit. The data are expressed as the mean ± SD. *p < 0.05; **p < 0.01 vs. parental.

Sorafenib is the only FDA-approved first-line drug for advanced HCC. However, many patients with HCC show resistance to sorafenib therapy. Therefore, the establishment of SR cells is urgently needed to study the mechanisms and new potential therapies in HCC. Recently, we established two SR HCC cell lines by chronic exposure to sorafenib treatment that was escalated from a low dose to higher doses. The human HCC cell lines HuH7 and Hep3B were incubated with sorafenib at a low dose (2.5 µM), and when the cells exhibited stable growth, we started to slowly enhance the dose of sorafenib (up to 10 µM, a clinically relevant dose). The medium containing sorafenib was renewed every 2~3 days. After 7 to 8 months, SR cells were obtained. Figure 1 shows that sorafenib inhibited the proliferation of parental Hep3B and HuH7 cells in a dose-dependent manner, whereas the SR cells (Hep3B-SR and HuH7-SR) were less sensitive to sorafenib. These data indicate that HuH7-SR and Hep3B-SR cells are significantly resistant to the cytotoxic effect of sorafenib.

### Compound 9a suppressed the growth of SR cells

**Figure 2:**
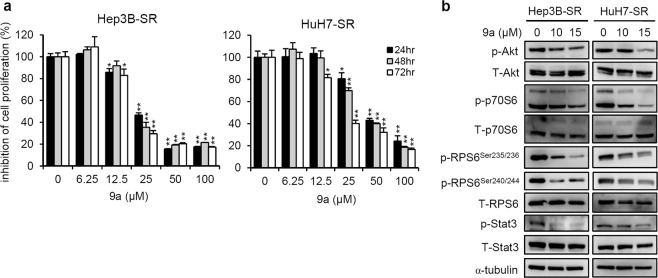
Anti-proliferative effect of compound 9a in sorafenib resistant liver cancer cells. **(a)** Hep3B-SR and HuH7-SR cells were exposed to DMSO control or 9a at the indicated doses for 24, 48 and 72 hrs, and cell proliferation was assessed using a MTT assay kit. *p < 0.05; **p < 0.01 vs. DMSO control. **(b)** Western blot analysis of Akt, p70S6, RPS6 and Stat3 phosphorylation in Hep3B-SR and HuH7-SR cells treated with 0, 10 and 15 µM of 9a for 48 hrs. The gels were cropped for conciseness. The original unedited pictures can be found in Suppl. Figure 1.

Next, we examined the cytotoxic effect of **9a** on SR cells. As shown in Fig. 2a, **9a** significantly inhibited the growth of Hep3B-SR and HuH7-SR cells in a dose-dependent manner. In Hep3B-SR cells, compound **9a** (25 µM) exhibited a 50% anti-proliferative effect; high doses of **9a** (50 µM and 100 µM) inhibited 80% of cell proliferation. In HuH7-SR cells, compound **9a** (25 µM) reduced cell proliferation by 20~30% after 24 or 48 hrs of treatment, and 72 hrs of treatment inhibited 60% of cell proliferation. A higher dose of **9a** (100 µM) inhibited 80% of HuH7-SR cell proliferation. These data suggest that compound **9a** may act as a novel agent to inhibit the proliferation of SR cells.

To study the mechanisms underlying the anti-proliferative effect of compound **9a** on both Hep3B-SR cells and HuH7-SR cells, Western blotting was performed and showed that compound **9a** inhibited Akt and p70S6 phosphorylation in Hep3B-SR and HuH7-SR cells (Fig. 2b). The downstream molecule RPS6 (at the Ser235/236 and Ser240/244 phosphorylation sites) was inhibited in **9a**-treated cells. In addition, the phosphorylation of Stat3 was inhibited in both **9a**-treated Hep3B-SR cells and **9a**-treated HuH7-SR cells (Fig. 2b).

### Treatment with compound 9a induced apoptotic signalling in SR cells

**Figure 3:**
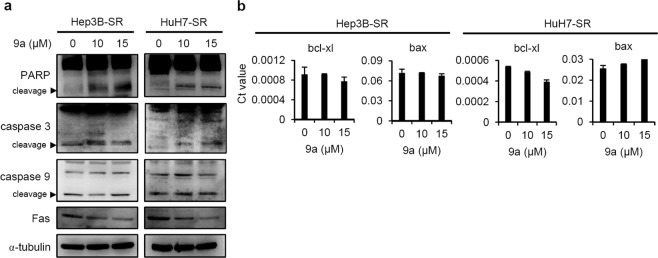
Effect of compound 9a on the apoptosis pathway in sorafenib resistant liver cancer cells. **(a)** Western blot analysis of PARP, caspase 9, caspase 3 cleavage, and Fas in Hep3B-SR and HuH7-SR cells treated with DMSO control and 9a (10 and 15 µM) for 48 hrs. The gels were cropped for conciseness. The original unedited pictures can be found in Suppl. Figure 2. **(b)** Q-PCR analysis of Bcl-xl and Bax expression in Hep3B-SR and HuH7-SR cells treated with DMSO control and **9a** (10 and 15 µM) for 24 hrs. The data are expressed as the mean ± SD.

Then, we studied whether compound **9a** decreases cell proliferation by triggering apoptotic signalling. As shown in Fig. 3a, **9a** treatment enhanced the cleavage of caspase 3 and PARP in Hep3B-SR and HuH7-SR cells and decreased Fas expression. The level of the cleaved form of caspase 9 did not increase in **9a**-treated cells. The mRNA expression of bax (a pro-apoptotic gene) and bcl-xl (an anti-apoptotic gene) was unchanged in **9a**-treated cells (Fig. 3b). These data indicate that the suppression of SR HCC cell proliferation in compound **9a**-treated cells arises primarily from blocking the AKT/p70S6 pathway and inducing caspase 3/PARP-dependent apoptotic signalling.

### Compound 9a suppressed the migration of SR cells

**Figure 4:**
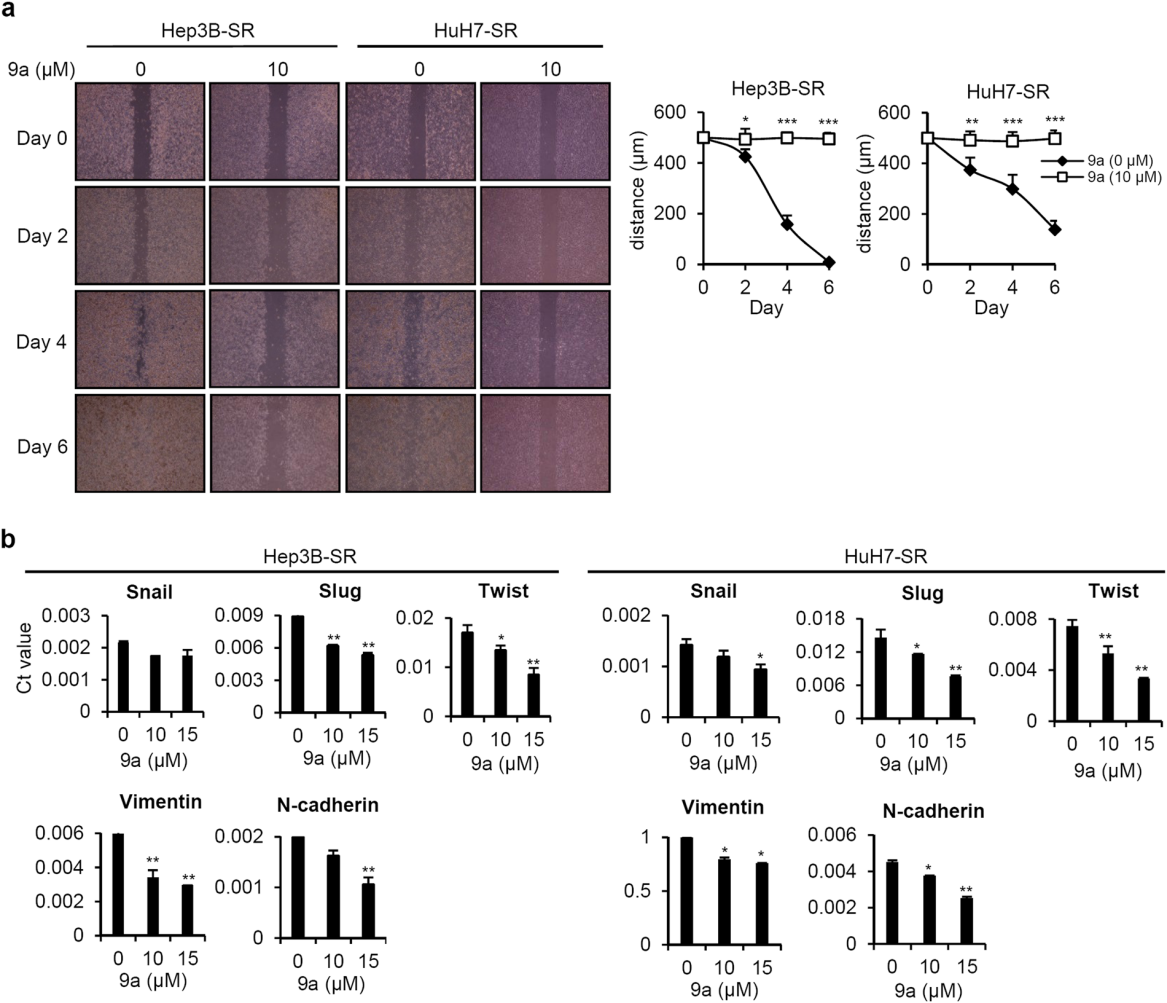
Anti-migration effect of compound 9a in sorafenib resistant liver cancer cells. **(a)** Hep3B-SR and HuH7-SR cells treated with vehicle control and 9a (10 µM) were grown in ibidi Culture Inserts. The images of the migrated cells in the gap area were taken and quantified at an indicated time point. **(b)** Q-PCR analysis of Snail, Slug, Twist, Vimentin, and N-cadherin expression in Hep3B-SR and HuH7-SR cells treated with DMSO control and 9a (10 and 15 µM) for 24 hrs. *p < 0.05; **p < 0.01; ***p < 0.001 vs. DMSO control. The data are expressed as the mean ± SD.

Epithelial-mesenchymal transition (EMT) is associated with chemoresistance in HCC^28,29^. After acquired sorafenib resistance develops, EMT promotes invasion and metastasis in HCC^30^. First, we assessed whether **9a** treatment affects the migratory activity of SR cells. As shown in Fig. 4a, SR cells exhibited migratory activities beginning on day2. Notably, **9a** treatment significantly inhibited SR cell migration. The expression of EMT-related transcription factors (snail, slug and Twist) and mesenchymal markers (vimentin and N-cadherin) was decreased in **9a**-treated cells in a dose-dependent manner (Fig. 4b). These data indicate that compound **9a** inhibits SR cell migration by repressing EMT-associated regulatory genes.

### Compound 9a administration overcame acquired sorafenib resistance in a xenograft model

**Figure 5:**
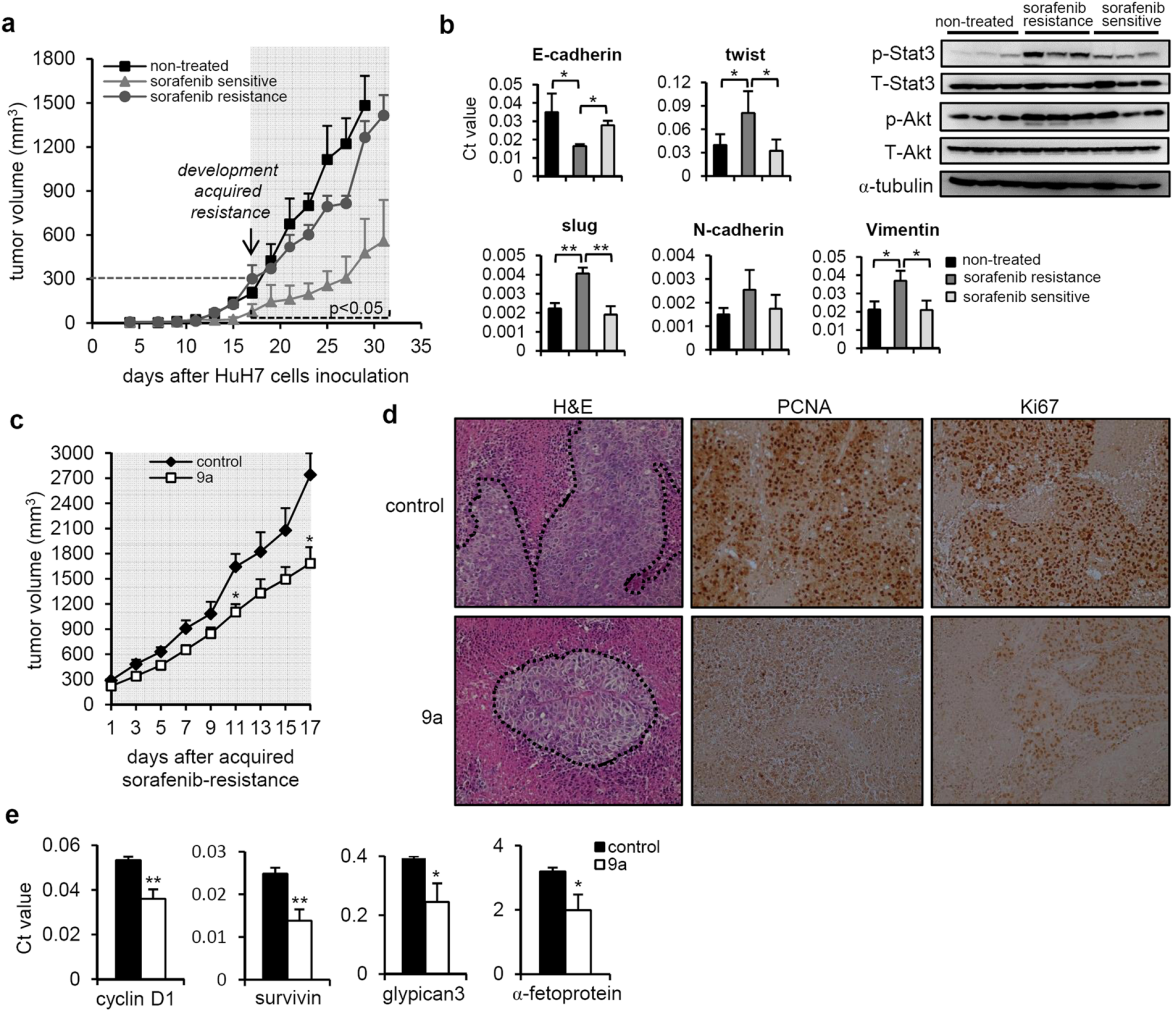
Therapeutic effects of compound 9a using acquired sorafenib resistance xenograft model. **(a)** Parental HuH7 cells were injected into NOD/SCID mice to initiate tumor growth and sorafenib was administrated when tumor size reached to 100 mm^3^. Sorafenib-treated tumor growth reveals into two patterns: sorafenib sensitive (triangular light gray line, n = 4) and lack of response to sorafenib (round dark gray line, sorafenib resistance, n = 5). Acquired resistance developed when tumor size reached to 300 mm^3^ (for at least 15 days) and significant statistical differences (p < 0.05) were observed between sorafenib resistant and sensitive tumor volumes in all lime points. The mean tumor volume ± s.e.m. at the indicated time is shown. **(b)** Q-PCR and Western blot analyses of E-cadherin, Slug, Twist, N-cadherin, Vimentin, p-Stat3 and p-Akt expression at the day 0 of compound 9a administration. **(c)** Effect of vehicle control and 9a administration on acquired sorafenib resistance xenograft tumor growth (n = 5 per group). The mean tumor volume ± s.e.m. at the indicated time is shown. **(d)** Representative IHC image of PCNA and Ki-67 staining in 9a treated acquired sorafenib resistance xenograft tumors. **(e)** Q-PCR analysis of cyclin D1, three human HCC markers (α-fetoprotein, survivin, and glypican 3) expression in 9a treated acquired sorafenib resistance xenograft tumors. *p < 0.05; **p < 0.01 vs. vehicle control.

To mimic HCC patients treated with sorafenib who then acquire sorafenib resistance, we established an HCC xenograft model of acquired resistance to sorafenib. Parental HuH7 cells were injected into NOD/SCID mice to initiate tumour growth, and sorafenib was administered when the tumour size reached 100 mm^3^. As shown in Fig. 5a, treatment of the mice with sorafenib significantly reduced the growth of HuH7 tumours (triangular light grey line, sorafenib sensitive); however, another group lacked a response to sorafenib (circular dark grey line, sorafenib resistant). Compared to the sorafenib-sensitive group, the SR group displayed rapid tumour growth when the tumour size reached 300 mm^3^ (p < 0.05), indicating that the tumours became resistant to sorafenib. Upregulation of the expression of p-Stat3, p-Akt and EMT markers (Fig. 5b), which have been reported to be characteristics of sorafenib resistance^30–32^, was observed in the SR tumours.

To evaluate whether compound **9a** potentiates an antitumour effect on an acquired sorafenib resistance xenograft model (Fig. 5a), we treated mice with **9a** when the tumour size reached 300 mm^3^. Although the tumour volumes in the **9a**-treated mice were similar to those in control mice for the first 9 days, we observed that the **9a**-treated mice had a decreased tumour growth rate after 11 days of treatment and eventually showed moderate growth inhibition at the end of the treatment period, indicating that compound **9a** administration overcame sorafenib resistance (Fig. 5b). In addition, the tumours from the **9a**-treated mice showed lower nuclear levels of immunostaining for Ki-67 and PCNA than those of the control mice (Fig. 5c), which was suggestive of inhibited proliferation in the SR tumours. On the other hand, the SR tumours from the mice treated with compound **9a** showed significantly lower levels of cyclin D1 and three HCC biomarkers (α-fetoprotein, survivin and glypican 3) than those from the vehicle control-treated mice (Fig. 5d).

## Discussion

Globally, HCC is one of the most common malignant tumours, and its incidence and mortality increase yearly^33,34^. Sorafenib, a multikinase inhibitor with anti-proliferative and anti-angiogenic properties, is the standard first-line clinical treatment for advanced HCC. Despite the capacity of sorafenib to increase the survival of HCC patients, the development of resistance to this drug has raised concerns in recent years^35^. When patients fail sorafenib therapy and have a poor prognosis, clinical trials of second-line drugs are necessary. Although a number of novel compounds (such as brivanib or everolimus) have been developed as second-line drugs^36,37^, they have failed in phase III studies. Regarding survival benefits or toxicity, none of these second-line drugs were superior to sorafenib. More recently, the FDA approved regorafenib, cabozantinib, ramucirumab, lenvatinib, and immune checkpoint inhibitors as second-line treatments for advanced HCC patients who fail to respond to sorafenib^38–41^. In 2017, the phase III placebo-controlled RESORCE trial showed that overall survival was extended from 7.8 months (in the placebo group) to 10.6 months (in the regorafenib group) after patients experienced disease progression while receiving sorafenib treatment, indicating the efficacy of regorafenib as a second-line drug for HCC^42^. However, only patients who developed Child-Pugh A status and tolerated sorafenib were recruited into the RESORCE study^42^. These limited criteria narrowed the pool of patients who required second-line treatment. In addition, the most common clinically relevant side effects, such as hypertension, hand-foot skin reaction, fatigue, and diarrhoea, were reported^42^. Regarding cost effectiveness, there was only a marginal increase in quality-adjusted life years for the high drug cost, which implied that regorafenib was not cost effective as a second-line drug for HCC treatment^43^. Therefore, the development of new compounds to improve the application of regorafenib as a new treatment for patients with advanced HCC or SR liver cancer is necessary.

**Figure 6:**
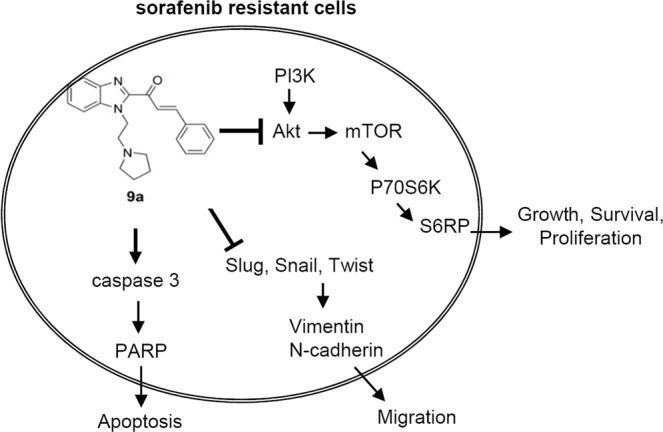
Schematic diagram of the signaling pathways involved in the inhibition of sorafenib resistance by compound 9a.

Previously, we identified a benzimidazole derivative bearing a pyrrolidine side chain (compound **9a**). We showed that combination treatment with compound **9a** plus sorafenib produced a more significant antitumour effect than treatment with either single agent alone^44^; however, whether **9a** overcomes sorafenib resistance is still unknown. The molecular pathogenesis of SR HCC involves different pathways and molecular aberrations^10,45^. Until now, the detailed mechanisms of acquired sorafenib resistance have not been thoroughly understood. Since 2013, microarray analyses have been used to analyse the possible mechanisms associated with sorafenib resistance in HCC^30,46^. Thus far, crosstalk involving PI3K/AKT, JAK/STAT, and EMT is the event most correlated with acquired sorafenib resistance in HCC^32,35^. In this study, we established SR cell lines and an acquired sorafenib resistance xenograft model. We further characterized the ability of compound **9a** to overcome sorafenib resistance both *in vitro* and *in vivo*. As shown in Fig. 6, compound **9a** may be a new anti-sorafenib resistance agent that interferes with the proliferation and migration of SR cells by inhibiting AKT/p70S6 and EMT signalling. In addition, caspase 3/PARP-dependent apoptotic signalling was induced in **9a**-treated cells.

The PI3K/Akt/mTOR pathway, an important cancer-promoting signalling cascade that regulates numerous biological functions, such as cell proliferation and migration, is activated in many types of human cancers, including HCC^47^. Sorafenib blocks the Ras/Raf/MAPK pathway and inhibits cell proliferation; however, sorafenib does not target the PI3K/AKT pathway^7^. PI3K/AKT activation plays an important role in mediating acquired resistance to sorafenib in HCC^48^. In the present study, we demonstrated that **9a** decreased the phosphorylation of AKT/p70S6/RPS6 and inhibited SR cell proliferation (Fig. 2). Chen *et al*. and Han *et al*. also reported that treatment with an AKT inhibitor suppressed the growth of SR tumours *in vivo*^48,49^. SHP-1 loss-dependent Stat3 activation is associated with the development of acquired resistance to sorafenib, indicating that targeting Stat3 is valuable approach for treating sorafenib resistance in HCC^50–52^. Our study also showed that compound **9a** significantly suppressed the phosphorylation of Stat3 in SR cells (Fig. 2). EMT is a developmental process that promotes invasion and metastasis in HCC through the loss of epithelial cell markers and enhancement of mesenchymal cell characteristics^53^. In SR HCC cells, the activation of the PI3K/AKT pathway is accompanied by EMT^30^, suggesting that sorafenib resistance mechanisms may involve EMT. Compound **9a** treatment significantly inhibited SR cell migration (Fig. 4a). In particular, the levels of EMT-related transcription factors and mesenchymal markers were decreased in **9a**-treated cells (Fig. 4b), indicating that the treatment effects of compound **9a** primarily arose from inhibiting the proliferation and migration of SR cells.

## Materials and Methods

### Benzimidazole derivative bearing a pyrrolidine side chain (compound 9a) and drug preparation

**Figure 7:**
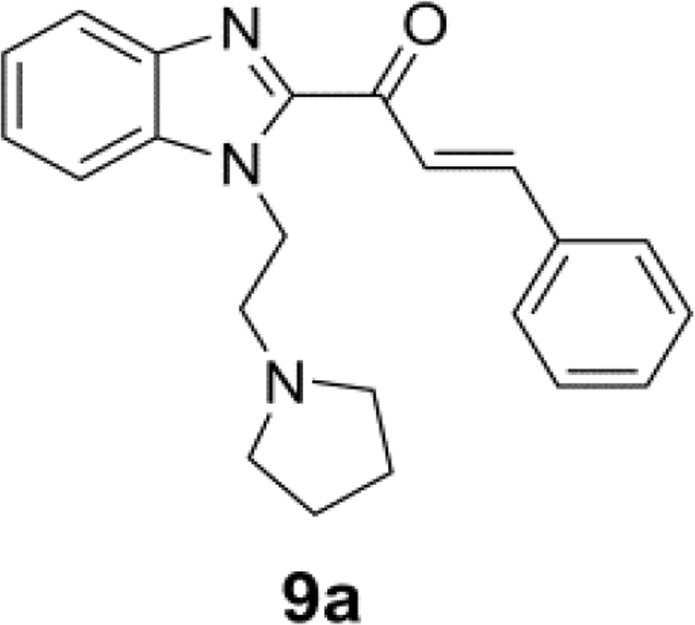
Structure of a benzimidazole derivative bearing a pyrrolidine side chain (compound 9a).

Figure 7 shows the structure of the benzimidazole derivative bearing a pyrrolidine side chain (compound **9a**). The detailed synthase of compound **9a** was described in our previous study^44^. Compound **9a** and sorafenib tosylate (ApexBio, purity >98%, Houston, TX, USA) were dissolved in DMSO and used at the concentrations indicated.

### Development of SR cells

Initially, the human HCC cell lines Hep3B and HuH7 were exposed to a low dose (2.5 µM) of sorafenib. When the cells exhibited stable growth, we started to increase the dose of sorafenib (5, 7.5, and then 10 µM). The sorafenib-containing medium was replaced every two days for 6 months. SR cells grew slowly in the medium containing 10 µM sorafenib (a clinically relevant dose) in further experiments^51^. The SR cells (Hep3B-SR and HuH7-SR) were routinely maintained under constant culture conditions including sorafenib exposure.

### Cell proliferation and migration assays

Cells (1.5 or 2.5×10^3^) were seeded in a 96-well plate. After the indicated treatments, the medium in each well was replaced with fresh DMEM containing 5 mg/ml 3-(4,5-dimethylthiazol-2-yl)-2,5-diphenyltetrazolium bromide (MTT) (Sigma-Aldrich, St. Louis, MO, USA). After 4 hrs of labelling, the medium was removed and replaced with 100 µl DMSO for 10 min at 37 °C. Samples were evaluated for absorbance at 540 nm. Culture inserts from Ibidi (Martinsried, Germany), which contained an approximately 500-μm cell-free gap after removing the culture insert, were used to compare cell migration between vehicle control- and compound **9a**-treated SR cells. In brief, the cells were grown to 90% confluence in the Ibidi culture inserts and allowed to attach overnight. After removing the culture inserts, images of the migrated cells in the gap area were acquired and quantified (ImageJ, National Institutes of Health, Bethesda, MD) at the indicated time points.

### Acquired sorafenib resistance xenograft model of HCC and compound 9a treatment

Since growing SR cells displayed a loss of cell-to-cell contacts and viable cells tended to easily detach from the surface and float in the culture dish^30^, it was difficult to cultured large numbers cells for injection into mice. We attempted to subcutaneously inject SR cells at a low cell number (1×10^5^) into NOD/SCID mice; unfortunately, we did not observe tumour growth in the 2.5-month follow-up period (data not shown). To mimic HCC patients treated with sorafenib who then acquired sorafenib resistance, we established an HCC xenograft model of acquired resistance to sorafenib. Six- to seven-week-old female NOD/SCID mice were purchased from the National Laboratory Animal Center, Taiwan. HuH7 cells (1×10^6^) were injected subcutaneously into the NOD/SCID mice. When the tumour size reached 100 mm^3^, the mice were assigned randomly to the vehicle control group or sorafenib treatment group (30 mg/kg/day, intraperitoneal injection). Tumour volume was monitored 3 times per week by using a Vernier calliper and then calculated as: (length × width^2^)/2. Upon development of sorafenib resistance (a ≥ 30% tumour volume increase in 3 days^46^ and a tumour size that reached 300 mm^3^ (for at least 15~17 days)), compound **9a** (4 mg/kg/day, intraperitoneal injection) or the vehicle control was administered. At the end of the experiments, the tumour masses were collected. Samples used for immunohistochemistry (IHC) staining were fixed in 10% formalin. The protocol was reviewed and approved by the Institutional Animal Care and Use Committee of Taipei Medical University (approval number: LAC-2016-0500) and carried out under the institutional and ARRIVE guidelines (https://www.nc3rs.org.uk/arrive-guidelines) for animal welfare standards.

### Western blot analysis

A lysis buffer supplemented with protease and phosphatase inhibitors was used to extract total protein from cultured cells. The protein concentration was measured by a protein assay dye (Bio-Rad Laboratories, CA), and all samples were normalized to 30 μg. Each cellular protein sample was separated by SDS-PAGE and transferred to a PVDF membrane. The following antibodies used in this study were purchased from Cell Signaling Technology (Beverly, MA, USA): anti-phospho- and anti-total-Akt, p70S6, RPS6, Stat3, anti-caspase 9, anti-caspase 3, anti-Fas and anti-PARP. Immunoblotting signals were normalized to the signal for total protein or α-tubulin (Sigma-Aldrich). Bands were visualized using an ECL detection reagent (Millipore Corporation, Billerica, MA, USA).

### RNA extraction, reverse transcription and real-time PCR

TRIzol Reagent (Ambion, Carlsbad, CA, USA) was used to isolate total RNA. Complementary DNA was produced from two micrograms of RNA using a High-Capacity cDNA Reverse Transcription kits (Applied Biosystems, Carlsbad, CA, USA). The specific primers are listed in Table 1. Real-time PCR was performed by using KAPA SYBR® FAST qPCR Master Mix (KAPA Biosystems, Boston, Massachusetts, USA) and analysed with the StepOne System (Applied Biosystems, Foster City, CA, USA). The cycle threshold (Ct) values were exported into Excel worksheets for analyses. The comparative Ct method was used to determine gene expression levels normalized to the level of GAPDH.

**Table 1:** Primers used for real-time PCR.

Gene	Forward Sequences (5′-3′)	Reverse Sequences (5′-3′)
Bcl-xl	GCTGCATTGTTCCCGTAGAG	GTTGGATGGCCACCTATCTG
bax	TGCTTCAGGGTTTCATCCAG	GGCGGCAATCATCCTCTG
Snail	GCTGCAGGACTCAATCCAGA	ATCTCCGGAGGTGGGATG
Slug	TGGTTGCTTCAAGGACACAT	GTTGCAGTGAGGGCAAGAA
Twist	CCCAACTCCCAGACACCTC	CAAAAAGAAAGCGCCCAAC
Vimentin	TGAAC GCAAAGTGGAATC	GTCAG GCTTGGAAACATC
N-cadherin	GGTGGAGGAGAAGAAGACCAG	GCATCAGGCTCCACAGT
Cyclin D1	AGGAACAGAAGTGCGAGGAGG	GGATGGAGTTGTCGGTGTAGATG
α-fetoprotein	CCCACTGGAGATGAACAGTCTTC	TGGCAAAGTTCTTCCAGAAAGG
Survivin	CGAGGCTGGCTTCATCCA	CAACCGGACGAATGCTTTTT
Glypican 3	GAGACTGCGGTGATGATGAAG	TCGGAGTTGCCTGCTGAC
GAPDH	TCACCACCATGGAGAAGGC	GCTAAGCAGTTGGTGGTGCA

### Immunohistochemical staining

Tumour tissue samples were fixed with 4% formaldehyde and dehydrated in a graded ethanol series and xylene. Paraffin-embedded sections (5 µm) were reacted with anti-Ki-67 and anti-PCNA antibodies (GenScript, Piscataway, NJ, USA) and detected by a Universal LSABTM2 kit (DakoCytomation Carpinteria, CA, USA).

### Statistical analysis

Non-parametric analysis was used for cell studies. Comparisons of two independent groups were performed with the Mann-Whitney U test. Parametric analysis was used for animal studies. Two-way analysis of variance was used for multigroup comparisons. Statistical analyses were performed using the SPSS program (SPSS Inc., Chicago, IL), and *p* < 0.05 was considered to indicate statistical significance.

## Supplementary information

supplementary info
