# Supplementary material for: Treatment with a new benzimidazole derivative bearing a pyrrolidine side chain overcomes sorafenib resistance in hepatocellular carcinoma
Source: Sci Rep. 2019 Nov 21;9:17259. doi: 10.1038/s41598-019-53863-2 (PMC6872581; doi:10.1038/s41598-019-53863-2)

Treatment with a new benzimidazole derivative bearing a pyrrolidine side chain overcomes sorafenib resistance in hepatocellular carcinoma

Fat-Moon Suk[1,2,#], Chao-Lien Liu[3,#], Ming-Hua Hsu[4], Yu-Ting Chuang[3], Jack P. Wang[5] and Yi-Jen Liao[3,*]

[1] Division of Gastroenterology, Department of Internal Medicine, Wan Fang Hospital, Taipei Medical University, Taipei, Taiwan.

[2] Department of Internal Medicine, School of Medicine, College of Medicine, Taipei Medical University, Taipei, Taiwan.

[3] School of Medical Laboratory Science and Biotechnology, College of Medical Science and Technology, Taipei Medical University, Taipei, Taiwan.

[4] Department of Chemistry, National Changhua University of Education, Changhua, Taiwan.

[5] Department of International Medicine, Taipei City Hospital Ranai Branch, Taipei, Taiwan.

[#] equal contribution.

* Correspondence: yjliao@tmu.edu.tw; Tel.: +886-2-27361661-3333

Supplementary Figure 1. Representative immunoblot of the original unedited pictures . The grouping of blots images were from different parts of the different gel. According to the different molecular weight of each protein, the gels were cropped and subjected to blot with different primary antibodies. The paired phospho- and total- protein was analyzed from the same sample. All samples were normalized to 30 μg of total protein.

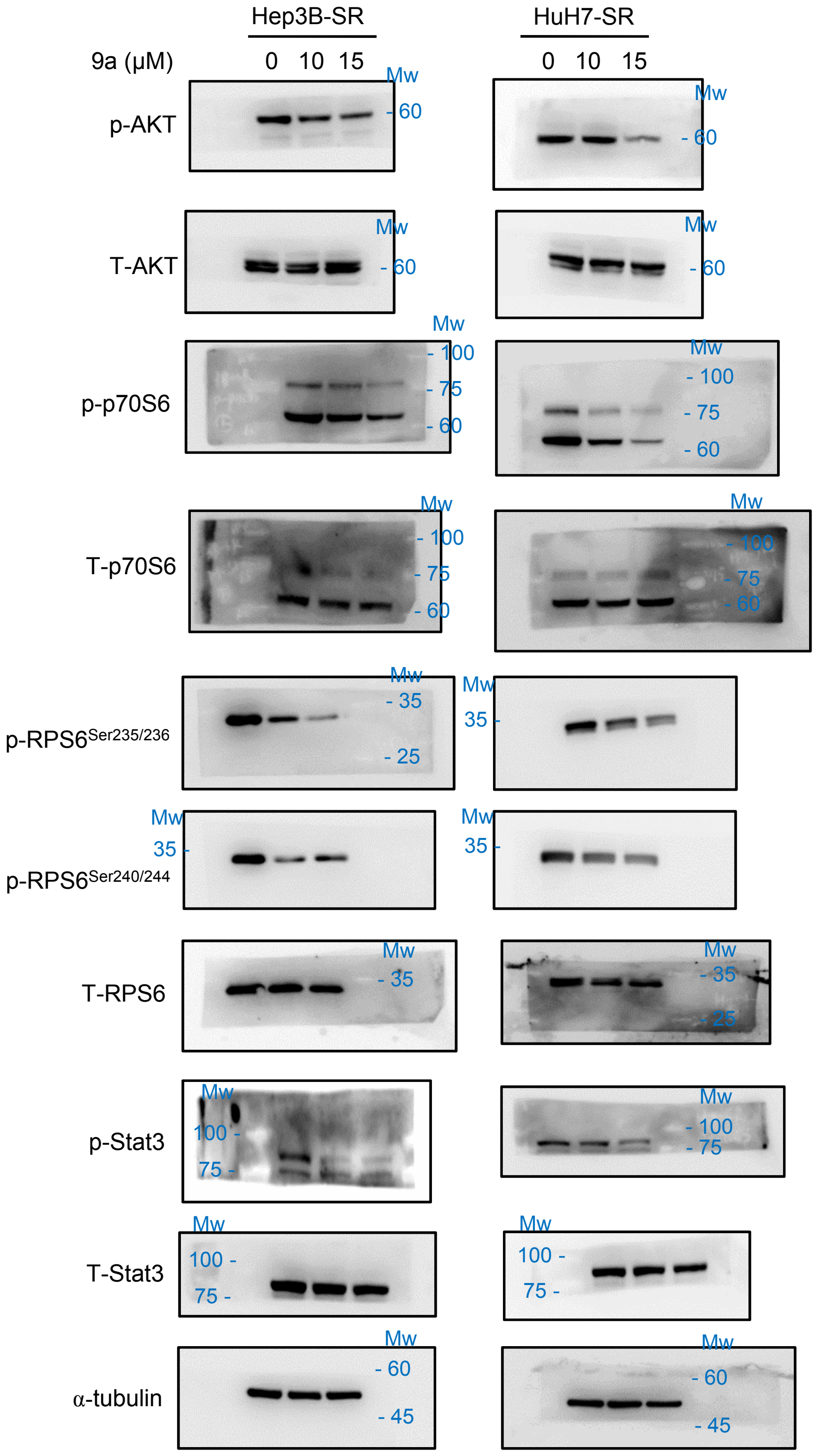

Supplementary Figure 2. Representative immunoblot of the original unedited pictures . The grouping of blots images were from different parts of the different gel. According to the different molecular weight of each protein, the gels were cropped and subjected to blot with different primary antibodies. All samples were normalized to 30 µg of total protein.

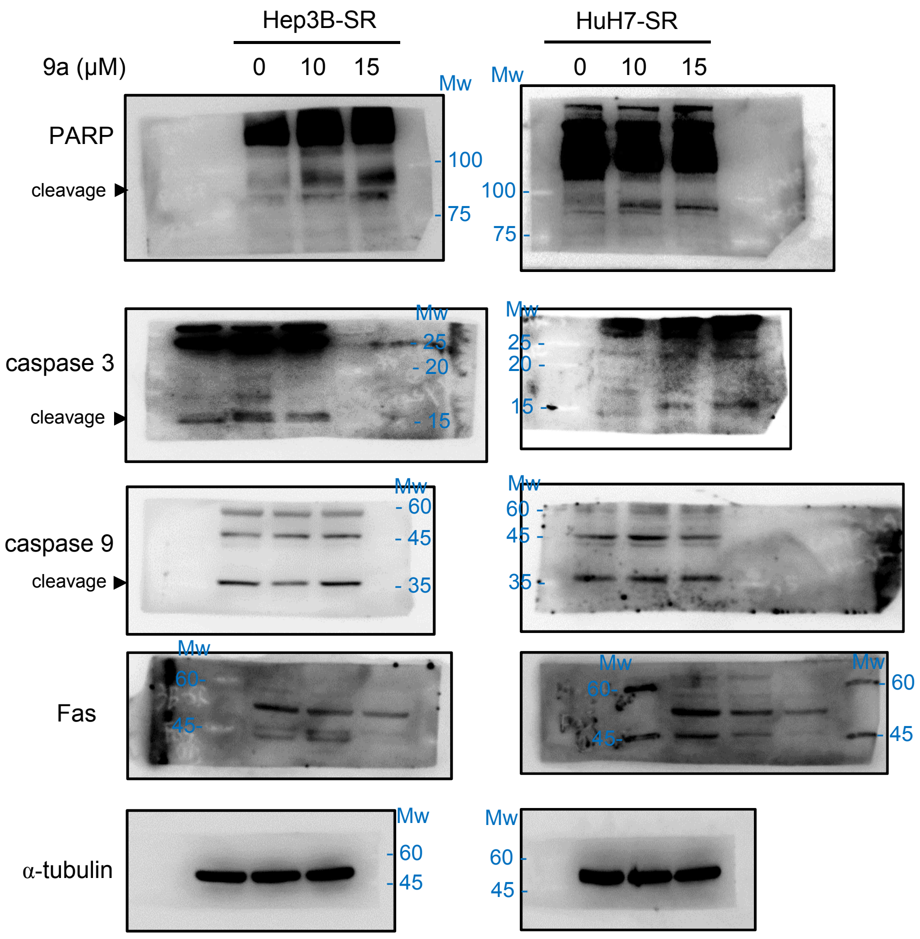

Supplement: Supplementary file 1 — supplementary info [file 41598_2019_53863_MOESM1_ESM.pdf]
